# Autologous Blood Transfusion Enhances Exercise Performance—Strength of the Evidence and Physiological Mechanisms

**DOI:** 10.1186/s40798-019-0204-1

**Published:** 2019-07-08

**Authors:** Sara Amalie Solheim, Jacob Bejder, Andreas Breenfeldt Andersen, Jakob Mørkeberg, Nikolai Baastrup Nordsborg

**Affiliations:** 1 Department of Nutrition, Exercise and Sport Sciences, University of Copenhagen, Universitetsparken 13, 2100 Copenhagen, Denmark; 2 Anti Doping Denmark, Brøndby, Denmark

**Keywords:** Phlebotomy, Transfusion, Arterial oxygen content, Maximal oxygen uptake, Maximal cardiac output, Anti-doping

## Abstract

This review critically evaluates the magnitude of performance enhancement that can be expected from various autologous blood transfusion (ABT) procedures and the underlying physiological mechanisms. The review is based on a systematic search, and it was reported that 4 of 28 studies can be considered of very high quality, i.e. placebo-controlled, double-blind crossover studies. However, both high-quality studies and other studies have generally reported performance-enhancing effects of ABT on exercise intensities ranging from ~70 to 100% of absolute peak oxygen uptake (VO_2peak_) with durations of 5–45 min, and the effect was also seen in well-trained athletes. A linear relationship exists between ABT volume and change in VO_2peak_. The likely correlation between ABT volume and endurance performance was not evident in the few available studies, but reinfusion of as little as 135 mL packed red blood cells has been shown to increase time trial performance. Red blood cell reinfusion increases endurance performance by elevating arterial oxygen content (C_a_O_2_). The increased C_a_O_2_ is accompanied by reduced lactate concentrations at submaximal intensities as well as increased VO_2peak_. Both effects improve endurance performance. Apparently, the magnitude of change in haemoglobin concentration ([Hb]) explains the increase in VO_2peak_ associated with ABT because blood volume and maximal cardiac output have remained constant in the majority of ABT studies. Thus, the arterial-venous O_2_ difference during exercise must be increased after reinfusion, which is supported by experimental evidence. Additionally, it remains a possibility that ABT can enhance repeated sprint performance, but studies on this topic are lacking. The only available study did not reveal a performance-enhancing effect of reinfusion on 4 × 30 s sprinting. The reviewed studies are of importance for both the physiological understanding of how ABT interacts with exercise capacity and in relation to anti-doping efforts. From an anti-doping perspective, the literature review demonstrates the need for methods to detect even small ABT volumes.

## Key Points

- Autologous blood transfusion increases exercise performance over a broad range of exercise intensities and durations.
- The exact relationship between performance increase and transfusion volume cannot be established due to a lack of studies. Even low-volume (135 mL) transfusions increase exercise performance.
- Autologous blood transfusion increases maximal oxygen uptake corresponding to the augmented haemoglobin concentration, whereas maximal cardiac output appears unaffected.

## Background

One of the first studies of how autologous blood transfusions (ABTs) affect exercise performance was completed in 1960 and demonstrated that sustainable power output at a heart rate of 170 bpm is impaired by ~ 8% after phlebotomy of ~ 600 mL and increases by ~ 4% after reinfusion of the refrigerated whole blood [1]. Since then, several studies have confirmed ABT to enhance exercise performance in various modes and durations [1–16]. However, ABT is associated with health risks (i.e. bacterial contamination, haemolysis, and allergic reactions) [17] and is against the spirit of sport. Therefore, the use of ABT is prohibited in competitive sports [18] and has been since 1986.

The hitherto-performed ABT studies have never been critically reviewed with a strong focus on research methodology, although the performance-enhancing effect of ABT appears well established and is in agreement with solid physiological principles. Important factors for study interpretation include number of participants, inclusion of controlled trials, blinding, transfusion volume, storage time, storage method, and exercise protocol. Considering the strengths and weaknesses of the existing studies is important not only for the physiological understanding of how ABT interacts with exercise capacity but also for anti-doping efforts, where knowledge of expected effects helps target and prioritize in-competition and out-of-competition testing. Thus, the present review aims to evaluate the magnitude of performance enhancement from various ABT procedures as well as to address the existing knowledge of the associated physiological mechanisms.

A systematic search for peer-reviewed publications was performed at http://www.pubmed.com using the following query: (((autologous blood transfusion) OR (red blood cell transfusion)) AND (performance OR exercise)). In addition, the retrieved reference lists were examined for unidentified studies. The literature search was terminated in January 2019. The exclusion criteria were studies published as abstracts only, studies on patients, and studies investigating the combination of ABT and high altitude acclimatization because altitude acclimatization may enhance performance [19]. In total, 28 research articles were included (Table 1).

**Table 1:** Summary of autologous blood transfusion (ABT) studies examining changes in aerobic power and endurance performance following ABT

Study	Participants (*n*), training status, and age (years; mean ± SD)	Study design	ABT volume of whole blood or equivalent when reinfused as packed RBCs (mL ^storage type, storage duration^)	Change in VO_2peak_	Performance test (duration/length)	Change in performance
Gullbring et al. [1]	6 M, UT, 34 ± 8	No control group	530–689^R, 1^	N/A	PWC/TTE cycling (N/A)	4%
Robinson et al. [20]	6 M, UT, 26 ± 6	No control group	1000–1200^R, 2^	↔	N/A	N/A
Ekblom et al. [13]	7 M, WT, 24 ± 1	No control group	800–1200^R, 4^	↑ 9% (1)	TTE running (~ 5 min)	↑ 23% (1)
Williams et al. [21]	20 M, MT-WT, 22 ± 4	Double-blind, placebo-controlled	500^R, 3^	N/A	TTE running (~ 10 min)	↔
Von Rost et al. [8]	6 M, MT, N/A	Controlled	900^R, 3–4^	↑ 6%	TTE running (~ 5 min)	↑ 37%
Ekblom et al. [22]	5 M, WT, 23–31	No control group	800^R, 5^	↑ 8%	N/A	N/A
Bell et al. [23]	15 M, MT, 18–24	Controlled	500^R, 3^	↔	TTE cycling (~ 15 min)	↔
Kots et al. [16]	10 M, UT-WT, 22–32	No control group	500^R, 3^	↔	PWC/TTE cycling (~ 10 min)	↑ 40%
Williams et al. [24]	16 M, WT, 32 ± 7	Double-blind, placebo-controlled	460^F, 3^	N/A	TTE running (~ 45 min)	↔
Buick et al. [10]	11 M, WT, 21 ± 3	Double-blind, placebo-controlled, crossover	900^F, 7^	↑ 5%	TTE running (7–10 min)	↑ 31–35%
Williams et al. [14]	12 M, WT, 33 ± 6	Double-blind, placebo-controlled, crossover	920^F, 7^	N/A	TT running (5 miles)	↑ 3%
Thomson et al. [25]	4 M, MT-WT, 23 ± 1	No control group	1000^F, 12^	↑ 12%	N/A	N/A
Thomson et al. [26]	4 M, MT-WT, 23 ± 1	No control group	1000^F, 12^	↑ 13%	N/A	N/A
Kanstrup and Ekblom [11]	5 M, WT, 25 ± 4	No control group	900^R, 5^	↑ 7%	TTE running (5–6 min)	↑ 24%
Robertson et al. [5]	9 W, UT-MT, 23 ± 2	Single-blind, placebo-controlled, crossover	475^F, 9^	↑ 10%	PWC cycling	↑ 23%
Celsing et al. [27]	9 M, WT, 29 ± 2	No control group	3500^R, F, 1–8^	↑ 19%	N/A	N/A
Spriet et al. [28]	4 M, WT, 25 ± 1	No control group	1350^F, 9–11^	↑ 7%	N/A	N/A
Muza et al. [29]	12 M, MT-WT, 27 ± 6	Double-blind, placebo-controlled	600^F, 6^	↑ 11%	N/A	N/A
Berglund and Hemmingsson [2, 30]	11 M, 1 W, WT, 19–35	Single-blind, placebo-controlled	1350 ^R, 4^	N/A	TT cross-country skiing (15 km)	↑ 5%
Brien and Simon [9]	6 M, WT, 24 ± 5	Double-blind, placebo-controlled, crossover	900^F, 11^	N/A	TT running (10 km)	↑ 3%
Celsing et al. [31]	8 M, UT-MT, 28 ± 3	Controlled	2250^F, 5–7^	↑ 7%	N/A	N/A
Sawka et al. [32]	9 M, MT, 30 ± 7	Double-blind, placebo-controlled	600^F, N/A^	↑ 11%	N/A	N/A
Ekblom and Berglund [12]	15 M, MT-WT, 27 ± 3	No control group	1350^F. 8^	↑ 8%	TTE running (7–9 min)	↑ 16%
Turner et al. [15]	7 M, MT, 26 ± 3	No control group	900^F, 8–12^	↑ 6%	TT cycling (30 min)	↑ 5%
Ziegler et al. [6]	8 M, UT-WT, 33 ± 2	No control group	450^R, 4^	↑ 5%	TT running (3 km)	↑ 5%
Malm et al. [3]	17 M, MT-WT, 34 ± 8	Placebo-controlled	900^F, 15–16^	↑ 12%	TTE running (6–7 min)	↑ 17%
Bennett-Guerrero et al. [4]	4 M, UT-MT, 34 ± 5 1 week, 29 ± 7 6 weeks	No control group	900^R, 1 or 6^	↑ 9% 1 week, ↑ 2% 6 weeks	TTE cycling (12–16 min)	↑ 8% 1 week, ↓3% 6 weeks
Bejder et al. [7]	9 M, WT, 29 ± 5	Double-blind, placebo-controlled, crossover	225^R, 4^ 675^R, 4^	N/A ↔	TT cycling (650 kcal)	↑ 4% ↑ 5%

*ABT* autologous blood transfusion, *M* men, *W* women, *VO*_*2peak*_ peak oxygen uptake, *RBCs* red blood cells, *R* refrigerated, *F* frozen, ***↑*** positive change, **↓** negative change, ↔ non-significant change/unchanged, *N/A* not available, *(1)* group 1, *WT* well-trained (VO_2peak_ of *>* 55 mL O_2_/kg/min), *MT* moderately trained (45–55 mL O_2_/kg/min), *UT* untrained (< 45 mL O_2_/kg/min), *TT* time trial, *TTE* time to exhaustion, *PWC* physical work capacity given as work (kg-force metres/min) performed to exhaustion

## Effect of ABT on Human Exercise Performance

### Intensity, Duration, and Training Status

The following section considers the importance of exercise intensity, exercise duration, and participants’ training status for the effect of ABT on human performance. In one of the first studies to address ABT’s effect on human exercise performance, time to exhaustion (TTE) lasting ~ 5 min increased 23% following reinfusion with 800 mL refrigerated packed red blood cells (RBCs) collected 4 weeks earlier [13]. The TTE was determined from treadmill running at a constant grade with a speed requiring maximal oxygen uptake (VO_2peak_). Likewise, TTE lasting ~ 6 min increased by ~ 25% compared to before collection when four participants received cold-stored packed RBCs from the sequential donation of 1200 mL of blood obtained ~ 30 days earlier [13]. However, the small sample size, the lack of a control group, and the non-blinded design preclude firm conclusions. When applying a double-blind, placebo-controlled crossover design and 7–11 weeks cryopreservation, performance was enhanced by ~ 30% in a TTE lasting ~ 10 min [10] and by 3% in 5 mile and 10 km time trials (TT) [9, 14] in well-trained males receiving a reinfusion of RBCs reconstituted to normal haematocrit (Hct) from a ~ 900 mL donation. Generally, the high-quality ABT studies report enhanced performance by ~ 3–5% compared with pre-reinfusion performance in time trials lasting ~ 30–45 min [7, 9, 14] and that TTE is prolonged by ~ 30% in a test lasting ~ 10 min [10]. It is of note that all the best controlled studies investigated well-trained male athletes (Table 1). Moreover, the performance enhancement was evident when evaluated a few days before and after reinfusion [1, 3–11, 13–16]. Importantly, both reinfusion of cryopreserved RBCs and reinfusion of cold-stored RBCs improve exercise performance irrespective of total haemoglobin mass recovery, which may be incomplete, especially in the context of cold storage, where ≤ 4 weeks elapse between donation and reinfusion [1, 6–8, 13, 30]. In support of the performance-enhancing effect of ABT, both high-quality and low-quality studies generally report that blood reinfusion improves exercise performance in tasks lasting from ~ 5 to ~ 45 min, which corresponds to exercise intensities of approximately 70 to ~ 100% of VO_2peak_. Furthermore, performance enhancement has been observed in both untrained and well-trained men (Table 1). Notably, the only study enrolling only women [5] also reported improved performance, but a lack of studies addressing a possible sex-specific effect is apparent. In relative terms, the effect of ABT in open-ended performance tests, i.e. TTE tests with a relatively short duration, appears superior, with an improvement range of 16–40%, to close-ended tests, i.e. TT tests of longer duration, with an improvement range of 3–5% (Table 1).

### ABT Volume and Physiological Effects

In the present section, it is considered whether a dose-dependent effect of ABT exists on maximal aerobic power and exercise performance.

#### Standard-Volume, High-Volume, and Low-Volume ABT

Reinfusion of cold-stored RBCs from 450–500 mL donations, herein termed ‘standard-volume ABT’, enhances 3 km treadmill time-trial performance by ~ 5% in moderately trained men [6] and TTE at maximal aerobic workload by ~ 40% in untrained men [16]. However, in some studies, reinfusion of 450 mL whole blood or 200 mL packed RBCs reconstituted with physiological saline to a normal Hct did not result in detectable performance gains in moderately trained to well-trained males, neither following cryopreservation [24, 28] nor after cold storage [21, 23]. Five of eight studies investigating reinfusion of a ‘standard-volume’ corresponding to ~ 500 mL reported improved performance, and the three negative findings must be interpreted cautiously (Table 1). There are several possible explanations why some studies have not observed an effect of a single standard-volume autologous reinfusion on VO_2peak_ or performance. First, the precision and random variation in the physiological measurements may mask performance-relevant increases. This explanation seems likely since inclusion of, for example, four subjects yields a detection limit for changes in absolute VO_2peak_ of ~ 6% when assuming a measurement error of 3% [33]. Moreover, Bell and co-workers [23] did not observe improved TTE, which appears to be likely related to variation in the small group of 5 subjects. Indeed, a numerical increase of > 20% in TTE was apparent in the ABT group, but unfortunately, no data on variation was reported for this outcome variable. Second, RBC storage lesions and degradation after reinfusion can be of importance since only ~ 70% (cold-stored) or ~ 50% (cryopreserved) of the haemoglobin (Hb) is preserved after storage [34]. Furthermore, ~ 25% of the infused RBCs can be degraded within 24 h [35], and a detectable effect of ABT on exercise performance immediately after reinfusion may disappear within a few days. In addition to variations in measures of physiological function and RBC storage lesions, low statistical power for determining subtle performance fluctuations and the selection of time points for performance evaluation are important. For instance, comparison of performance before blood donation and after reinfusion separated by 21 days [21] introduces variation over time as well as a risk of insufficient haemoglobin mass recovery [6, 36]. Indeed, when performance was evaluated prior to phlebotomy and after the reinfusion 21 days later, 10 of 15 participants performed 4–5% worse [21]. However, this finding appears more likely to be related to an insufficient total haemoglobin (tHb) recovery period and natural fluctuations in exercise capacity than lack of effect of ABT on performance. Clearly, variation in measurements, physiological ability, RBC storage lesions, and RBC degradation as well as study design impact the outcome of studies evaluating the effect of standard-volume ABTs on exercise performance.

The same study design factors as outlined above affect studies of ABT with larger volumes, but the expected higher ‘signal-to-noise’ ratio reduces the risk of not identifying meaningful effects. However, when interpreting studies of ABT volumes of ~ 900 mL or more, which is the case for 17 of the studies listed in Table 1, it must be considered whether the associated increase in blood viscosity lowers maximal cardiac output (Q_max_) more than increased arterial oxygen content (C_a_O_2_) can compensate for [28] or if it even reduces tissue perfusion. Compromised Q_max_ appears of little relevance because an autosomal dominant erythrocytosis carrier (i.e. increased sensitivity to EPO) with a haematocrit of more than 60% is also an endurance sport Olympic gold medallist [37]. With regard to tissue oxygen delivery, O_2_ consumption and contractile power of blood-perfused canine gastrocnemius muscles are compromised only when haematocrit is higher than 60%, despite reduced absolute blood flow [38]. In support of the above observations, exercise performance in rodents is optimal at a haematocrit of approximately 60% [39]. Thus, even markedly elevated blood viscosity after high-volume ABT does not counteract the performance-enhancing effect. This notion is supported by the high-quality studies of ABT volumes ~ 900 mL reporting improved exercise performance in humans [9, 10, 14].

The possible performance-enhancing effects of ABTs of less than 450 mL, termed ‘low-volume ABT’ in the present review, have not yet been fully scientifically addressed (Table 1), even though dishonest athletes are suspected to reinfuse low (≤ 150–200 mL) volumes [40]. To our knowledge, we recently completed the first study on the effect of a low-volume ABT using a randomized, double-blind, placebo-controlled crossover design. We demonstrated that reinfusion with ~ 135 mL of cold-stored RBCs, corresponding to ~ 225 mL of whole blood, is sufficient to increase mean power by ~ 5% and reduce elapsed time by ~ 4% in a 650 kcal cycling TT [7]. Concomitantly, haemoglobin concentration ([Hb]), Hct, and RBC count increased by 3% compared to their levels 3 days before reinfusion. Hence, even low ABT volumes improve exercise performance.

#### Is There an ABT Dose-Response Relationship?

Only a few studies have directly addressed whether ABT volume is decisive for the magnitude of effect on absolute VO_2peak_ and/or exercise performance. One of the earliest studies to investigate the dose-dependency of ABT on VO_2peak_ applied stepwise reinfusions separated by 2–7 days [28]. Each reinfusion consisted of freeze-preserved RBCs, reconstituted with physiological saline to a normal Hct, from 450 mL whole blood harvested 9–11 weeks earlier [28]. The direct effect on endurance performance was not evaluated, but the primary outcome, VO_2peak_, surprisingly remained unchanged following the first reinfusion in the four well-trained endurance runners. VO_2peak_ did, however, increase non-significantly (*p* > 0.05) by ~ 200 mL O_2_/min (4%) in all four subjects after the second reinfusion, and an additional significant increase to ~ 340 mL O_2_/min (7%) above control was observed after the third reinfusion [28]. Thus, an undetected dose-dependent response may have been apparent. This interpretation is supported by the gradual increase in VO_2peak_ observed with four successive reinfusions of 450 mL [31]. Thus, the impact of ABT on VO_2peak_ apparently follows a dose-response relationship, as could be expected, although a well-designed study to address this question is lacking.

#### Does Storage Technique Impact ABT’s Effect on Exercise Performance?

Two main techniques are used for RBC storage: refrigeration (cold storage) or freezing (cryopreservation). In the clinical setting, cold-stored RBCs must be reinfused after 35–42 days, depending on the storage solution, due to increasing storage lesions, whereas cryopreservation allows for storage for up to 30 years [41]. Cold-stored blood appears to be the least probable strategy used by dopers because 20–59 days is required for full tHb restoration after an ~ 550 mL phlebotomy [42] and the effect on [Hb], and thus performance, may be counteracted by suppressed haemoglobin mass at the time of reinfusion. On the other hand, pre-reinfusion handling of cryopreserved blood is associated with a 20% larger loss of the initially removed Hb compared with that of refrigerated blood due to the necessary freeze-thaw-wash process following cryopreservation [34]. Moreover, cryopreservation is more expensive and technically challenging than cold storage. No studies have directly compared the impact of storage technique on performance. However, it is important to note that the evaluation of ABT’s effect on exercise capacity requires that the performance evaluation be completed a few days before and after reinfusion, independent of storage technique. Evidently, both storage techniques are able to enhance aerobic performance (Table 1), although the shortcomings of the existing protocols need to be acknowledged.

#### Study Design Considerations

Several factors influence the interpretation of ABT and exercise performance studies. These factors include storage technique, number of participants, inclusion of control trials, blinding procedure, transfusion volume, storage time, exercise protocol, and statistical analyses. From a human performance and anti-doping perspective, the lack of a statistically significant difference following a treatment is of limited value if just one participant gains a competitive advantage. The margin of difference between competitors is small, and a < 1% effect is of relevance. However, such small changes are difficult to detect in a scientific study. A useful approach in this context could be the evaluation of clinical significance or relevance by reporting effect sizes and minimal clinically important differences [43]. However, the sensitivity issue appears to be of less importance in ABT studies, as a performance-enhancing effect of several percent is present, even when low-volume transfusions are applied [7]. To date, six studies with endurance performance measurements have applied a double-blind, placebo-controlled design with a total of 6–20 participants [7, 9, 10, 14, 21, 24]. All except two [21, 24] of these studies reported improved performance after the reinfusion of autologous blood, which may be ascribed to a sample size of 5 [21] or 8 [24] in each group or variation in the measurements.

## Physiological Mechanisms Responsible for the Performance-Enhancing Effect of ABT

Theoretically, endurance performance is related to VO_2peak_ relative to body weight and the % of VO_2peak_ that can be sustained during prolonged intense endurance exercise (i.e. 30–40 min for a 10-km run). However, how does ABT alter VO_2peak_ and the ability to sustain a high percentage of VO_2peak_? In the following sections, the physiological mechanisms that may explain the performance-enhancing effect of ABT are considered.

### ABT Increases Maximal Muscular Oxygen Delivery

Maximal O_2_ delivery to the contracting limb muscles can be augmented via increased limb blood flow, increased C_a_O_2_, or both. An increase in maximal limb blood flow requires increased Q_max_ or altered blood flow distribution. In the ‘Is Maximal Cardiac Output Increased by ABT?’ and ‘Is Maximal Limb O_2_ Delivery and Extraction Increased by ABT?’ sections, ABT’s potential effect on Q_max_, limb blood flow, and C_a_O_2_ is addressed.

#### Is Maximal Cardiac Output Increased by ABT?

When maximal exercise with a large muscle mass is performed in normoxia, Q_max_ determines the upper limit for O_2_ delivery to the active muscle groups and thereby becomes the limiting factor for VO_2peak_ in endurance-trained, untrained, and generally healthy individuals [44]. Hence, increasing Q_max_ will increase an individual’s maximal oxygen uptake and thereby also maximal endurance performance by allowing the individual to exercise at a higher absolute workload, while metabolism remains primarily aerobic [45]. In cross-sectional studies, total blood volume and tHb are tightly associated with VO_2peak_, whereas [Hb] is not [46]. In agreement with this idea, Q_max_ is closely related to VO_2peak_ [47], and thus, it may be hypothesized that an increased blood volume can increase Q_max_ and thereby performance. Indeed, a training-induced expansion of total blood volume appears to be the primary cause of increased VO_2peak_ via increased Q_max_ [48]_._ Thus, it is tempting to suggest that the ABT-induced increase in tHb increases VO_2peak_ by increasing Q_max_. However, it is important to note that the close association between absolute VO_2peak_ and tHb when determined in a mixed group of humans is caused primarily by differences in size, including ventricular mass and total blood volume, which defines Q_max_ [47], whereas no close association is evident in a homogenous group of athletes [49]. With respect to the effect of blood reinfusion on VO_2peak_, it is important that total blood volume alterations appear to depend on the time of investigation after reinfusion. Total blood volume appears unaffected ≥ 24 h post-reinfusion of 800 to 2250 mL of refrigerated whole blood [22] as well as refrigerated [50] or freeze-preserved [31, 32, 50] RBCs reconstituted with physiological saline to a normal Hct. In contrast, 1 h after reinfusion of packed RBCs from 900 mL of whole blood, a 5% increase in total blood volume occurs together with increases in Hct (2%), [Hb] (4%), VO_2peak_ (7%), and cycling performance lasting 3–6 min (24%) [11]. As such, total blood volume may be unaffected by ABT when determined more than 24 h after reinfusion due to a rapid compensatory reduction in plasma volume. However, the small number of studies on ABT and total blood volume and the variances and challenges associated with blood volume determination preclude a firm conclusion.

Reinfusion of autologous blood acutely increases central venous pressure [20], which usually increases Q_max_ due to an enlarged end-diastolic volume and thereby causes a higher stroke volume and Q_max_, according to the Frank-Starling mechanism [51]. However, it is likely that the increased central venous pressure after blood reinfusion is transient (< 24 h) and related to the acute volume expansion, but this remains to be investigated. The most direct approach to determine the effect of ABT on Q_max_ is to measure Q_max_. A few studies have directly evaluated Q_max_ after blood reinfusion. In 7 males who received RBCs obtained from 900 mL donations 2–3 months earlier, no difference existed approximately 24 h after reinfusion in the invasively determined Q_max_ of ~ 22 L/min, despite a clear ~ 3% increase in VO_2peak_ [15]. In 9 women, reinfusion of RBCs from two 450 mL phlebotomies, conducted 9 and 17 weeks prior, did not alter the Q_max_ of ~ 17 L/min, as indirectly determined 48 h later by CO_2_ rebreathing [5]. Moreover, in four male endurance runners, the reinfusion of RBCs from 900–1350 mL blood 3–4 weeks after phlebotomy increased VO_2peak_ but only non-significantly increased dye dilution-determined Q_max_ from ~ 28 to ~ 33 L/min approximately 2 days later [28]. Thus, Q_max_ appears unaffected even by reinfusion of rather large volumes and cannot explain the increase in VO_2peak_ and performance after ABT. In this context, it must be noted that determination of Q_max_ is technically difficult and associated with variance; therefore, a moderate undetected effect of ABT on Q_max_ may exist.

#### Is Maximal Limb O_2_ Delivery and Extraction Increased by ABT?

Limb blood flow can be as high as 2000–3000 mL/min per kg of muscle [52], and the muscular oxygen uptake capacity is ~ 600 mL/kg/min in healthy subjects, which is much higher than the amount of O_2_ supplied during whole-body exercise [53]. Hence, it is evident that limb blood flow is limited by Q_max_ during intense whole-body exercise. Thus, if maximal limb blood flow during intense whole-body exercise is increased by reinfusion, this would increase maximal aerobic energy production and thereby performance. However, maximal limb blood flow appears unlikely to increase since Q_max_ appears unaffected by reinfusion, as highlighted in the ‘Is Maximal Cardiac Output Increased by ABT?’ section. In this context, it must be noted that, for example, brain blood flow is primarily dependent on C_a_O_2_ [54], and therefore, the ABT-induced increase in arterial oxygen content may reduce blood flow to other tissues and organs. As a consequence, it remains a possibility that more blood is redistributed to the exercising muscles and that maximal limb blood flow is marginally increased by RBC reinfusion despite unaltered Q_max_. However, this hypothesis remains to be investigated.

Reinfusion of autologous blood is usually [7, 10, 13, 14, 28, 31, 32], but not always [3], accompanied by elevated Hct and/or [Hb] compared to the pre-reinfusion levels. Accordingly, reinfusion increases the O_2_ carrying capacity per litre of blood, and this has been highlighted in previous reviews as the main physiological mechanism causing enhanced performance following ABT [55]. Indeed, stepwise reinfusions of four blood bags, in eight untrained to moderately trained men, resulted in VO_2peak_ increases of approximately 40 mL O_2_/min (1%), 130 mL O_2_/min (3%), 230 mL O_2_/min (5%), and 320 mL O_2_/min (7%) and increases in [Hb] of approximately 4%, 7%, 12%, and 13% from the first to the last reinfusion, respectively [31], as mentioned in the ‘Is There an ABT Dose-Response Relationship’ section. This result resembles the findings of Spriet and co-workers [28] and corresponds to an approximately 0.47% increase in VO_2peak_ per 1% increase in [Hb] (*R*^2^ = 0.97) [31]. For the ABT-induced increase in [Hb] to augment VO_2peak_ without affecting Q_max_, the arterial-venous O_2_ difference (a-v O_2_ difference) across the exercising muscle groups must be increased. The ABT-induced increase in C_a_O_2_ and concurrent unchanged [15, 25] or reduced [22] mixed venous O_2_ content (C_v_O_2_) strongly suggest an increased extraction [15] after reinfusion, but the small sample sizes (*n* = 4–7) preclude a firm conclusion.

### ABT, Anaerobic Energy Production, and Metabolic Acidosis

#### Blood Lactate Response to Exercise

Increased maximal capacity for oxygen delivery likely reduces the anaerobic energy contribution at intensities above ~ 70% of VO_2peak_, as illustrated by reduced plasma lactate concentrations ([La]) after ABT measured at the same absolute workload [5]. The reinfusion of RBCs packed saline obtained from phlebotomy of 900 mL and 1350 mL whole blood causes a marked reduction in blood lactate values following exercise at 91% of VO_2peak_ (from ~ 6 mM before reinfusion to ~ 2 mM after) and after maximal treadmill running at 100% of control VO_2peak_ (from ~ 12 mM before to ~ 5 mM after), concomitant with less disturbed arterial acid-base status 5 min after exercise [28]. Furthermore, blood reinfusion reduces the lactate concentration by approximately 50% in runners when determined 4 min after submaximal (~ 70% VO_2peak_) treadmill running [10]. In five trained subjects, venesection increased the lactate response to exercise at ~ 70% of VO_2peak_ from 6.0 to 7.6 mM, while reinfusion reduced lactate to 4.6 mM [22]. This reduction in plasma-borne markers of anaerobic energy contribution during submaximal exercise may increase the %VO_2peak_ defining the lactate threshold, which is an important determinant of endurance performance [56, 57]. Since most endurance competitions are not performed at intensities corresponding to 100% of VO_2peak_, this may be the most important performance-enhancing effect of ABT. However, it should also be acknowledged that some parts, e.g. the final part of a 5000-m or 10,000-m race, a cycling stage with mountain climbs or the like, are likely to elicit VO_2peak_.

#### Blood pH Response to Exercise

During intense exercise, anaerobic energy production results in increased plasma hydrogen concentration ([H^+^]) [58]. Metabolic acidosis is immediately counteracted by hyperventilation, bicarbonate buffering, and non-bicarbonate buffering. The relationship between changes in plasma lactate concentration and pH is indicated by the in vivo buffer capacity: ∆[La^-^]/∆pH [59]. RBCs are primary extracellular buffers, and therefore, the total blood buffer capacity depends on the total RBC volume and the expression of RBC membrane-bound transport systems, such as the AE1 isoform of the anion-exchange transporter [60, 61] and the MCT1 isoform of the monocarboxylate transporter (La^−^/H^+^ co-transporter) [61], as well as the enzyme cytoplasmic carbonic anhydrase, which catalyses the reversible hydration of carbon dioxide (CO_2_); CO_2_ + H_2_O (water) ⇋ H_2_CO_3_ (carbonic acid) ⇋ HCO_3_^−^ (bicarbonate) + H^+^ [60, 61]. Hence, the RBCs added through ABT have the potential to theoretically improve systemic pH regulation. However, the possible effect is small since a low-volume ABT expands the total RBC volume by only a few percent. Nevertheless, the relevance for performance in elite sports with small margins may exist, but this remains to be investigated.

## Possible Influence of ABT on Anaerobic Performance and Repeated Sprints?

The reinfusion of autologous blood increases the maximal muscular oxygen uptake, which is the product of limb blood flow and arterial-venous O_2_ difference. Blood reinfusions might also increase performance in short, anaerobic, intermittent exercises. Since the aerobic energy provision increases when sprints are repeated [62, 63] and the aerobic capacity is important for recovery between sprints [64], it appears likely that repeated sprint ability is improved by blood reinfusion. [La^−^] in blood and muscle increases progressively during a 4 × 30 s all-out air-braked cycle test in moderately trained males [65]. Additionally, muscle La^−^ accumulates non-linearly during a 2 × 30 s all-out cycle test at a work load corresponding to 0.075 × kg of body weight [66] and during 3 × 30 s all-out isokinetic cycling at 100 rpm in healthy, recreationally active subjects [58]. The highest impact of blood reinfusion on repeated sprint ability is thus likely to occur in the later sprints. In support of this notion, the level of aerobic ATP provision during sprints has been found to increase progressively as work duration increases [67, 68] and as sprints are repeated [62, 63]. Additionally, the potentially improved blood buffer capacity during polycythaemia might contribute to the extracellular pH defence against lactic acidosis, as outlined in the ‘ABT, Anaerobic Energy Production, and Metabolic Acidosis’ section [59, 61]. Furthermore, the nature of the recovery between sprints is likely of importance for repeated sprint ability. For example, active recovery is advantageous for the mean power output of sprints when repeated Wingate anaerobic tests are performed [62]. The O_2_ availability for mitochondria is known to be decisive for creatine phosphate recovery [69], and an ABT-induced increase in C_a_O_2_ thus appears likely to improve recovery between sprints. Supporting this theory, the 11% increase in [Hb], following recombinant human EPO administration, reduces the accumulation of blood La^−^ and plasma hypoxanthine after fifteen 6-s high-intensity uphill running sprints with 24 s of passive rest [70]. Interestingly, in the only study to investigate the effect of ABT on repeated cycling sprint ability, we observed no change in performance after reinfusion, despite an ~ 9% increase in tHb and [Hb] [7]. The combined effect of the reinfusion of ~ 135 and ~ 235 mL cold-stored RBCs, corresponding to approximately 675 mL whole blood, did not alter 4 × 30 s all-out cycling performance, either when determined as the mean power of all sprints or when evaluated as the mean power in each sprint. The two reinfusions were performed within ~ 24 h, and the performance was evaluated a few days before the first reinfusion and after the last reinfusion, separated by 8 days, as opposed to before the phlebotomy and after the reinfusion. This allowed for the evaluation of ABT-induced changes, although the haematological parameters had not returned to the pre-phlebotomy level at the time of reinfusion. The results suggest that reinfusion with ~ 370 mL of packed RBCs does not affect repeated sprint ability as measured by 4 × 30 s of all-out cycling interspersed with 4 min of rest. However, the effect of ABT in other repeated sprint modalities and the effect of larger ABT volumes are unknown.

Overall, the literature is scarce regarding the effects of ABT on repeated sprint performance. Nevertheless, the aforementioned potential effects of ABT in enhancing O_2_ delivery and blood buffer capacity might be reflected in improvement in fatigue resistance during repeated sprints and thereby enhanced repeated sprint performance in some repeated sprint modalities.

## Conclusion

The literature review revealed that ABT enhances performance across a span of exercise intensities from ~ 70 to 100% of VO_2peak_ and durations of 5–45 min, and these results were also observed in well-trained athletes. The impact of ABT on VO_2peak_ apparently exhibits a dose-response relationship, but well-designed studies of likely associated gradual performance effects are lacking. However, reinfusion with as little as 135 mL packed RBCs increases time-trial performance. Red blood cell reinfusion increases endurance performance by elevating C_a_O_2_. The increased C_a_O_2_ is accompanied by increased VO_2peak_ as well as reduced lactate concentrations, i.e. reduced anaerobic energy contribution at submaximal intensities. Both effects improve endurance performance, the latter because most endurance competitions are not performed at intensities corresponding to 100% of VO_2peak_. Apparently, the magnitude of change in haemoglobin concentration explains the increase in VO_2peak_ associated with ABT because blood volume and maximal cardiac output remain constant in the majority of ABT studies. Thus, the arterial-venous O_2_ difference during exercise must be increased after reinfusion, which is supported by experimental evidence. Additionally, it remains a possibility that ABT can enhance repeated sprint performance, but studies are lacking. The only available study did not observe a performance-enhancing effect of reinfusion on 4 × 30 s sprinting.

The findings are of importance for both the physiological understanding of how ABT interacts with exercise capacity and in relation to anti-doping efforts. From an anti-doping perspective, the literature review demonstrates the need for analytical methods able to detect even minor blood manipulations.

## Data Availability

Not applicable. The data that support the findings of this review are available from the cited research articles.

## Abbreviations

[Hb]: Haemoglobin concentration
[La^-^]: Lactic acid concentration
ABT: Autologous blood transfusion
a-v O_2_ difference: Arterial-venous O_2_ difference
C_a_O_2_: Arterial oxygen content
CO_2_: Carbon dioxide
C_v_O_2_: Mixed venous oxygen content
EPO: Erythropoietin
H^+^: Hydrogen ion
H_2_CO_3_: Carbonic acid
H_2_O: Water
Hb: Haemoglobin
HCO_3_^−^: Bicarbonate
Hct: Haematocrit
La^−^: Lactic acid
Q_max_: Maximal cardiac output
RBC: Red blood cell
tHb: Total haemoglobin mass
TT: Time trial
TTE: Time to exhaustion
VO_2peak_: Peak oxygen uptake
